# The Discriminative Power of Different Olfactory Domains in Parkinson's Disease

**DOI:** 10.3389/fneur.2020.00420

**Published:** 2020-06-02

**Authors:** Yuwen Zhao, Yan He, Runcheng He, Yangjie Zhou, Hongxu Pan, Xiaoting Zhou, Liping Zhu, Xun Zhou, Zhenhua Liu, Qian Xu, Qiying Sun, Jieqiong Tan, Xinxiang Yan, Beisha Tang, Jifeng Guo

**Affiliations:** ^1^Department of Neurology, Xiangya Hospital, Central South University, Changsha, China; ^2^Department of Geriatrics, Xiangya Hospital, Central South University, Changsha, China; ^3^Center for Medical Genetics, School of Life Sciences, Central South University, Changsha, China; ^4^Key Laboratory of Hunan Province in Neurodegenerative Disorders, Central South University, Changsha, China; ^5^National Clinical Research Center for Geriatric Disorders, Changsha, China

**Keywords:** Parkinson's disease, Sniffin' sticks, olfactory dysfunction, Chinese population, olfactory domains

## Abstract

**Background and Purpose:** Olfactory dysfunction is one of the most common non-motor symptoms in Parkinson's disease (PD) preceding the motor symptoms for years. This study aimed to evaluate different olfactory domains in PD patients in comparison with healthy controls and to explore the relationships among olfactory deficit and other clinical manifestations in patients with PD.

**Methods:** Sniffin' Sticks test, which detects olfactory threshold, discrimination, and identification (TDI), were conducted in 500 PD patients and 115 controls. Furthermore, demographic and clinical data including motor and other non-motor symptoms were collected.

**Results:** In the single olfactory model, the identification test showed the area under the receiver operating characteristic (ROC) curve (AUC = 0.818), followed by threshold test (AUC = 0.731) and discrimination test (AUC = 0.723). Specifically, the identification test has a similar discriminative power as the TDI score (0.818 and 0.828, respectively, *p* = 0.481). In the integrated olfactory model involved with other non-motor manifestations, identification test scores performed as good as the TDI score in differentiating PD patients from controls (0.916 and 0.918, respectively, *p* = 0.797). In PD patients, age and cognition together explained 7.5% of the variance of the threshold score, while age, cognition, and gender accounted for the 15.2% explained variance of the discrimination score, while cognition, age, the ability of daily living, and gender together interpreted 11.1% of the variance of the identification score.

**Conclusion:** Our results indicated that the identification domain was the most practical olfactory factor in differentiating PD patients, and the combination of several different manifestations was better than a single symptom. Furthermore, the olfactory identification score may be associated with the ability of daily living.

## Introduction

The olfactory deficit is one of the most important non-motor symptoms that could appear to precede motor symptoms in Parkinson's disease (PD) (1–4). Olfactory dysfunction has been incorporated both in Movement Disorder Society clinical diagnostic criteria for PD and research criteria for prodromal PD, demonstrating its role in the diagnosis and prediction of PD (5–7). PD-associated smell dysfunction involves several domains of odor perception, i.e., detection threshold, identification, discrimination, and memory (8–10). The structural changes in the olfactory bulb, neurotransmitter system dysfunction, and inflammatory activity in the brain are all possible mechanisms of olfactory impairment in PD (11).

In terms of differentiating PD from control subjects, some studies have shown that the sensitivity and specificity of olfactory testing are better than other biomarkers, including single-photon emission computed tomography (SPECT) and positron-emission tomography (PET) imaging of the dopamine (DA) transporter (12). In PD patients, different odor domains have relatively uniform impairment (13–15); however, data on the magnitude of different odor domains impairment and its ability in distinguishing PD from healthy control remains insufficient. Mahlknecht and colleagues investigated the power of olfactory function in distinguishing PD with a proper sample size, but the olfactory test was limited to the identification domain (16). Krismer and colleagues researched different olfactory domains, but the sample size is relatively small (17). Studies have indicated that combining olfactory tests and other prodromal non-motor features could recognize the risk of PD more efficiently (18); however, similar studies have never been conducted in Chinese PD populations.

In some studies, considered as an independent feature of PD, the olfactory deficit was not found to have significant associations with other symptoms of the disease (19). However, Mahlknecht and colleagues suggested that olfactory dysfunction may facilitate the development of PD from associated with rapid eye movement sleep behavior disorder (RBD) (20). There were still inconsistent conclusions about the relationship between olfactory function and other clinical manifestations in PD (21, 22).

In this study, we comprehensively evaluated the discriminative power of different olfactory domains, as well as in condition of combining other non-motor symptoms for early diagnosis of Chinese PD patients, and explored the potential relationship between olfactory deficit and other motor or non-motor features in Chinese PD patients. The aim was to identify the specific olfactory domain that has the best discriminative power and to ascertain if the olfactory deficits were independent features of PD.

## Methods

### Participants

All the PD patients were recruited from the inpatients and outpatients of the Department of Neurology of Xiangya Hospital, Central South University, Hunan, China, between September 2014 and July 2017 at Parkinson's Disease & Movement Disorders Multicenter Database and Collaborative Network in China (PD-MDCNC, http://pd-mdcnc.com:3111/). Patients with idiopathic PD were diagnosed by no less than two experienced neurologists according to the United Kingdom Parkinson's Disease Brain Bank criteria (23). Healthy controls without neurological diseases were recruited from Health Management Centers of Xiangya Hospital. Participants with a history of respiratory system diseases, nasal or sinonasal diseases, and neurological or sinonasal surgery were excluded. The Medical Ethics Committee of Xiangya Hospital approved the study, and the participants gave informed consent for the investigation.

### Assessments

Demographic data of all subjects were collected including gender, age, years of education, smoking status, and family history of PD. Seven domains of non-motor symptoms were evaluated by the Non-Motor Symptom Scale (NMSS) (24, 25), including cardiovascular, sleep/fatigue (26), mood, perceptual problems, gastrointestinal, urinary, and sexual issues. Cognitive functions were evaluated by the Mini-Mental State Examination (MMSE) (27, 28). Olfactory function was evaluated by the Sniffin' Sticks test.

In addition, age at onset, course of disease, and anti-PD medication were recorded for patients. Motor functions were evaluated by the Unified Parkinson's Disease Rating Scale (UPDRS) and Hoehn Yahr Scale (H-Y). In addition, tremor score was measured by adding up scores of tremors at rest and action and postural tremor of hands from the UPDRS score, while bradykinesia score was calculated by score on finger taps, hand movements, rapid alternating movements of hands, and leg agility. Rigidity score was added up by the scores on rigidity of the neck, hands, and feet (29). Disease motor subtype (30) was classified as tremor-dominant (TD) phenotype when the ratio of tremor score and postural instability and gait difficulty (PIGD) score was no <1.5, whereas patients with a ratio of no more than 1.0 were defined to PIGD phenotype, and rest of patients belonged to the indeterminate phenotype. UPDRS is made up of four sections. Of them, UPDRS part II is characterized by questionnaires about self-evaluation of the activities of daily life, including speech, swallowing, handwriting, dressing, hygiene, falling, salivating, turning in bed, walking, and cutting food. UPDRS part III was used to assess motor ability. A higher UPDRS score means more severe symptoms. A higher MMSE score means better cognitive condition. A higher NMSS score means more severe non-motor symptoms. Dyskinesia was affirmed by experienced neurologists (31).

### Sniffin' Sticks

Sniffin' Sticks test consist of three parts, and they were tests for olfactory threshold, discrimination, and identification domain. Threshold and discrimination tests were conducted in the condition of subjects' eyes closed or blindfolded to prevent them from recognizing through the color of pen caps.

Both threshold and discrimination tests comprised 16 triplets' pens (total of 48 pens) numbered from 1 to 16. The color of three pen caps differed from each other, which are red, blue, and green. Identification tests were comprised of 16 common odors, each of which presented 4 alternative odors to choose from. Odor threshold test could evaluate the ability to perceive the lowest concentration of an odorant by the subject, odor discrimination test measured the ability to differentiate two different odors, and odor identification test measured the ability to perceive and name the presented odor out of four alternative answers (32). The threshold score (*T*-score) equals the mean of the last four of seven scores, while the discrimination score (*D*-score) and identification score (*I*-score) equals the numbers of correct responses, respectively (33). The threshold, discrimination, and identification (TDI) score equals to the total score of three tests. The cutoff of the TDI score was 30.3 for ages from 16 to 35 years, 27.3 for ages from 36 to 55 years, and 19.6 for subjects older than 55 years, according to the standard of Hummel et al. (34). A higher score means better olfactory perception.

### Statistics

All data were not normally distributed by the Kolmogorov–Smirnov test. All continuous variables were described as median and interquartile range (IQR), such as age, years of education, disease duration, UPDRS II, UPDRS III, MMSE, NMSS scores, and so on, while the categorical variables were described as a percentage, such as a gender, smoking status, dyskinesia status, and so on.

To establish which of the olfactory test is of service for differentiating PD patients from healthy controls, we calculated the receiver operating characteristic (ROC) curves for each of the olfactory tests separately and for any two or three tests combined. The single binary logistic regression models were developed with diagnosis as the dependent variable: using age, years of education with threshold score; then age, years of education with discrimination score; next age, years of education with identification score; and then age, years of education with any two or three of olfactory domain score added together. Afterward, integrated binary logistic regression models were developed with diagnosis as the dependent variable: using the above variable with each model combining other non-motor features, including MMSE and NMSS (cardiovascular, sleep/fatigue, mood, perceptual problems, gastrointestinal, urinary, and sexual issues). We graphed ROC curves with sensitivity and specificity estimates and corresponding area under the ROC curve (AUC), as well as positive likelihood ratios (LR+), negative likelihood ratios (LR–), positive predictive values (PPV), and negative predictive values (NPV). The ROC cutoffs were chosen when Youden's Index to get the maximum value. We compared AUC between TDI score single model and other single models by MedCalc software, as well as in the integrated models.

To compare demographic information and clinical features between PD with hyposmia and PD with normosmia, we used the analysis of chi-square tests for measurement data and non-parametric tests for continuous data.

To explore the contribution of different variables to the olfactory score, we used four stepwise multiple linear regression analyses (methods = stepwise, F-to-enter = 0.05, F-to-remove = 0.1). In the multiple linear regression analysis, independent variables include demographic factors (age, sex, educational years, smoking status), motor clinical symptoms (disease duration, UPDRS II points, UPDRS III points, dyskinesia), and other clinical symptoms (MMSE, NMSS). We did stepwise multiple linear regression analyses with threshold score, differentiation score, identification score, and TDI score as dependent variables, respectively.

Data were analyzed using SPSS version 18. *p* < 0.05 were considered significant.

## Results

### Demographic and Clinical Characteristics

In total, we recruited 500 patients (male, 269, 53.8%) with a median age at assessments of 60 years and a median age at onset of 55 years. The 115 healthy controls (male, 50, 43.5%) have a median age of 55 years. Median disease duration of PD was 3 years, whereas the median UPDRS II and UPDRS III scores were 12 and 26, respectively (Table 1).

**Table 1:** Basic information and motor features in patients with Parkinson's disease (PD) and controls.

**Items**	**Patients with PD (*n* = 500)**	**Normal controls (*n* = 115)**
Sex (male %)	269 (53.8%)	50 (43.5%)
Age (year)	60 (52–67)	55 (49–64)
Educational years (year)	9 (6–12)	9 (9–12)
Smoking or not	144 (28.8%)	32 (27.8%)
Age of onset (year)	55 (47–62)	–
Duration(year)	3 (2–6)	–
UPDRS II	12 (9–17)	–
UPDRS III	26 (19–38)	–
H-Y stage	2 (1.5–3)	–

*Data for continuous variables are presented as medial levels (IQRs)*.

### Olfactory Test Alone

Of the 500 included patients, 343 patients had hyposmia, whereas 157 patients had a normal sense of smell, according to the standard of Hummel's (34). The median TDI score of PD patients was 19.50 and that of the control subjects was 28.25. Median threshold, discrimination, and identification scores of PD patients were 4.75, 7, and 7, respectively, while control subjects were 7.50, 10, and 10, respectively. After age and years of education correction, every single olfactory score of PD patients were significantly lower than controls subjects (all *p* ≤ 0.001), as well as the total TDI score (Table 2).

**Table 2:** Olfaction function in patients with Parkinson's disease (PD) and controls.

**Items**	**Patients with PD (*n* = 500)**	**Normal controls (*n* = 115)**	*p-*value^*^
Threshold score (T)	4.75 (2.25–7.00)	7.50 (5.50–9.25)	0.001
Discrimination score (D)	7 (5–9)	10 (8–11)	<0.001
Identification score (I)	7 (5–9)	10 (9–12)	<0.001
TD score	12.50 (8.50–15.75)	16.75 (14.50–19.50)	<0.001
TI score	11.87 (7.75–15.50)	17.75 (15.50–20.75)	<0.001
DI score	14 (11–18)	20 (17–23)	<0.001
TDI score	19.50 (14.25–24.25)	28.25 (24.50–31.00)	<0.00

* *p-value was calculated after adjustment of age and educational years*.

**Figure 1:**
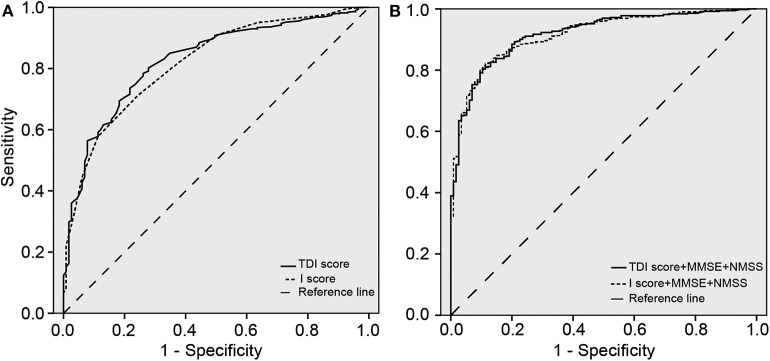
Receiver operating characteristic (ROC) curves. **(A)** Relating sensitivity and specificity for olfactory threshold, discrimination, and identification (TDI) and I scores in differentiating Parkinson's disease (PD) patients from healthy controls. **(B)** Relating sensitivity and specificity for olfactory TDI and I scores combining Mini-Mental State Examination (MMSE) and Non-Motor Symptom Scale (NMSS) in differentiating PD patients from healthy controls.

ROC curves of the TDI and I scores were drawn by SPSS (Figure 1). Every model had diagnostic value between these two groups (all *p* < 0.05). AUC of different olfactory domains and their sensitivity, specificity, LR+, LR–, PPV, and NPV in single olfactory models and integrated models were reported (Table 3).

**Table 3:** Area under the receiver operating characteristic curve (AUC) of different olfactory domains and their sensitivity, specificity, LR+, LR–, PPV, and NPV in single olfactory models and integrated models.

**Models**	**Olfactory tests**	**Sensitivity**	**Specificity**	**PPV**	**NPV**	**LR+**	**LR–**	**AUC**	***p*1-values**	***p*2-values**
(A) SINGLE MODELS^*^										
	TDI score	0.802	0.722	0.891	0.259	2.885	0.274	0.828	<0.05	
	TD score	0.692	0.748	0.884	0.235	2.746	0.412	0.772	<0.05	<0.05
	TI score	0.796	0.730	0.894	0.262	2.948	0.279	0.819	<0.05	0.306
	DI score	0.738	0.748	0.893	0.251	2.929	0.350	0.815	<0.05	0.239
	T score	0.612	0.783	0.888	0.229	2.820	0.496	0.731	<0.05	<0.05
	D score	0.544	0.791	0.875	0.215	2.603	0.576	0.723	<0.05	<0.05
	I score	0.712	0.757	0.894	0.247	2.930	0.380	0.818	<0.05	0.481
(B) INTEGRATED MODELS^**^										
	TDI score	0.803	0.896	0.971	0.515	7.721	0.220	0.918	<0.05	
	TD score	0.846	0.835	0.956	0.559	5.127	0.184	0.902	<0.05	<0.05
	TI score	0.838	0.870	0.965	0.556	6.446	0.186	0.913	<0.05	0.298
	DI score	0.783	0.913	0.975	0.495	9.000	0.238	0.913	<0.05	0.374
	T score	0.756	0.887	0.966	0.459	6.690	0.275	0.890	<0.05	<0.001
	D score	0.707	0.922	0.975	0.424	9.064	0.318	0.886	<0.05	<0.001
	I score	0.813	0.896	0.971	0.528	7.817	0.209	0.916	<0.05	0.797

*p1-values show the significance of differentiating PD patients from controls calculated by SPSS. p2-values were calculated by MedCalc software. p2-values of (A) were calculated by comparing AUC between each score with TDI score in single models, p2-values of (B) were calculated by comparing AUC between each score with TDI score in integrated models. p < 0.05 were considered significant*.

* *Single models were built upon each corresponding olfactory test, after adjustment of age and educational years*.

** *Integrated models were built upon each corresponding olfactory test and MMSE, NMSS, after adjustment of age and educational years*.

*PPV, positive predictive values; NPV, negative predictive values; LR+, positive likelihood ratios; LR–, negative likelihood ratios; T, threshold; D, Discrimination; I, Identification*.

In single models, the TDI score (AUC = 0.828) and identification score (AUC = 0.818) were better than threshold score (AUC = 0.731) and discrimination score (AUC = 0.723) at differentiating PD patients from controls. By comparing AUC between identification score with TDI score in single models, there was no significant difference of discriminative power between TDI and identification scores (difference between areas = 0.01, *z* statistic = 0.706, *p* = 0.481).

### Olfactory Test Combining Other Non-motor Features

Compared to control subjects, PD patients had poorer performance on other non-motor features, including cognitive, cardiovascular, sleep/fatigue, emotional, perceptual problems, gastrointestinal, and urinary and sexual dysfunction. These integrated models were much better than the corresponding single olfactory models (Figure 1; Table 3). Similarly, with a combination of other non-motor features mentioned above, the TDI score (AUC = 0.918) and the identification test score (AUC = 0.916) were slightly better than threshold score (AUC = 0.890) and discrimination score (AUC = 0.886) at differentiating PD patients from controls. Identification and TDI scores have no significant difference of discriminative power (difference between areas = 0.002, *z* statistic = 0.257, *p* = 0.797).

### Olfaction in PD Patients

Of the 500 included PD patients, 343 (68.6%) patients had hyposmia. The median TDI scores for the hyposmia and normosmia groups were 16.50 and 24.75 points, respectively. Median threshold, median discrimination, and median identification scores of the normosmia group were 7.25, 9, and 9. By contrast, those of the hyposmia group were 3.50, 6.5, and 6 (Table S1).

Compared with the normosmia group, we observed that patients in the hyposmia group were significantly more often men, with fewer educational years, more severe rigidity symptom, and more severe cognitive problems (*p* < 0.05).

Finally, stepwise multiple linear regression analysis removed confounding factors whose *p* ≥ 0.05. By multiple regression analysis, the final models interpreted 16.0% of variance in TDI score (*p* < 0.001, *R*^2^ = 0.160, Table S2a), 7.5% of the variance in threshold score (*p* < 0.001, *R*^2^ = 0.075, Table S2b), 15.2% of the variance in discrimination score (*p* < 0.001, *R*^2^ = 0.152, Table S2c), 11.1% of the variance in identification score (*p* < 0.001, *R*^2^ = 0.111, Table S2d). The variance inflation factor (VIF) showed no evidence of a multicollinearity problem among the independent variables. Older age, lower MMSE scores, and male sex were significantly associated with lower TDI scores. Older age and lower MMSE scores were associated with lower threshold scores. Older age, lower MMSE scores, and male sex were significantly associated with lower discrimination scores. Lower MMSE, older age, higher UPDRS II score, and male sex were associated with lower identification scores.

## Discussion

In our study, first of all, we confirmed olfactory deficit in PD, including the impairment of olfactory threshold, discrimination, and identification ability. Meanwhile, the olfactory identification test distinguished best between PD patients and control subjects among three olfactory tests in the single or integrated models.

Previous studies have compared different olfactory domains in discriminating patients with PD and control subjects, as well as other neurodegenerative diseases. For instance, Berendse et al. (35) supported that odor identification was better in differentiating patients with PD from control subjects than the odor discrimination task. Then, the same group (14) supported that a combination of an olfactory threshold test and a 16-item olfactory identification test scored the best in sensitivity and specificity in discriminating between PD patients and controls. A meta-analysis (36) once concluded that the olfactory threshold test should be included in the test for subclinical patients with PD. Hummel et al. (37) reported that PD patients performed relatively well in the olfactory threshold task, while they perform poorly in olfactory discrimination and identification compared to other diseases, such as sinonasal disease, postinfectious and posttraumatic status, and so on.

In the background of these published studies, our current study had resembled but more detailed implications. In our study, a combination of olfactory identification and discrimination tests could not improve the diagnostic accuracy of a single olfactory identification test, which was partly in accordance with other studies (14, 35). However, combining three olfactory tests did slightly improve the diagnostic value, which still supported that olfactory deficit was based on the dysfunction of multiple olfactory domains (38).

However, besides PD, the olfactory deficit was also the feature of other causes (39). Therefore, we usually combine other non-motor manifestations to distinguish between PD patients and controls. In our integrated model of differentiation, no matter which single or combined odor tests were chosen to represent for olfactory function, olfactory dysfunction was always included in the model. In summary, we may believe that the olfactory test was an essential part of the PD clinical studies, especially in a large scale of screening of PD or in PD modeling establishment. It was consistent with the study of Antje et al. (40) that the combination of olfactory tests and other tests may constitute a screening tool for PD.

When the entire three olfactory tasks represented olfactory function, its corresponding integrated model had the highest AUC based on the corresponding ROC curve, whereas the AUC of an integrated model constructed by odor identification was not significantly lower. Therefore, in large scales of studies containing an olfactory evaluation of patients with PD, we may believe that the olfactory identification test was sufficient enough to represent olfactory function in an integrated model to differentiated PD patients from controls subjects. After all, the entire olfactory test was more time and energy consuming. In large-scale studies, it can save researchers' and patients' time and energy to accomplish other necessary non-motor manifestations in the integrated model.

According to the standard of olfactory dysfunction (34), patients with olfactory deficit were more often men, had fewer educational years, and presented more severe cognitive problems. Liu et al. (41) also supported that male patients had significantly more deficits in olfaction than female patients. It may also suggest that not only the age of subjects but also gender and educational years should be considered into the future standard of olfactory deficits.

Moreover, in the olfactory threshold and discrimination domains, other clinical manifestations did not remain in their corresponding regression models. It indicated that olfactory threshold and discrimination domains were independent features of PD, just like tremors (42), which were not clearly related to other PD manifestations. We found a lack of relationship between dyskinesia and olfactory function in PD, which was consistent with the conclusion of Stephenson et al. (43) that there was no significant effect of olfactory performance on the risk of motor complications, such as falls and dyskinesia. This result indicated that olfactory threshold and discrimination deficit developed and progressed before the development of motor symptoms and maintained throughout the process of the disease (44). However, in the olfactory identification domain, the UPDRS II score was included in its regression model except for age, gender, and MMSE score, which partly resembled the observations in other studies that disease stage explained part of the variance in olfactory discrimination score of PD patients (13, 35). The lesser UPDRS II scores were associated with higher identification scores, which indicated that the odor identification task is associated with the activities of daily living. As previous studies pointed out, the performance of daily activities can be limited and conditioned by non-motor symptoms (45). An alternative explanation for the association between the odor identification and daily activities, but not UPDRS III, is methodological, since the former section is mainly based on a patient/caregiver self-completed questionnaire, whereas UPDRS III is based on professional rating (46). Therefore, it deserves further replication in larger cohorts in the future.

In conclusion, our study showed that the odor identification domain can basically represent olfactory functions in discriminating PD patients from controls, suggesting a specific aspect of one symptom may be an adequate representation of this certain symptom, which was energy and time saving especially in data collection of a large cohort study. Our data also indicated that the combination of different kinds of symptoms would be better in discriminating PD than a single symptom. Furthermore, the olfactory threshold and discrimination domains were independent features of PD, while worse daily living ability was associated with lower olfactory identification scores.

## Data Availability Statement

All datasets generated for this study are included in the article/Supplementary Material.

## Ethics Statement

The studies involving human participants were reviewed and approved by the Medical Ethics Committee of Xiangya Hospital. The patients/participants provided their written informed consent to participate in this study. Written informed consent was obtained from the individual(s) for the publication of any potentially identifiable images or data included in this article.

## Author Contributions

YZha, YH, JG, BT, and QS designed the experiments. YZha, YH, RH, YZho, XiZ, XuZ, LZ, ZL, QX, JT, and XY collected clinical data and performed phenotype analyses. YZha, YH, and HP analyzed the data and reference management. YZha, YH, and JG wrote and revised the manuscript. All authors contributed to read and approved the submitted version.

## Conflict of Interest

The authors declare that the research was conducted in the absence of any commercial or financial relationships that could be construed as a potential conflict of interest.
