# Supplementary material for: The Discriminative Power of Different Olfactory Domains in Parkinson's Disease
Source: Front Neurol. 2020 Jun 2;11:420. doi: 10.3389/fneur.2020.00420 (PMC7280480; doi:10.3389/fneur.2020.00420)
Supplement: Supplementary file 1 [file Table_1.docx]

**Table S1. Demographic, Motor and Non-motor Manifestations in Patients with PD**

Data for continuous variables are presented as medial levels (IQRs). Values in bold refer to statistically significant differences (*P* < 0.05). Memory and smell of patients were assessed with MMSE and Sniffin’s Sticks respectively.

^a^*P* values were calculated with Mann-Whitney.

^b^*P* values were calculated with the Pearson χ^2^ tests.

| **Items** | **PD with Hyposmia**  **(n = 343)** | **PD with Normosmia**  **(n = 157)** | ***P* value** |
| --- | --- | --- | --- |
| **Sex (male %)** | 196 (57.1%) | 73 (46.5%) | **0.033^b^** |
| **Age (y)** | 59 (51-67) | 61 (56-67) | 0.143**^a^** |
| **Education years(y)** | 9 (6-12) | 9 (7-12) | **0.043^a^** |
| **AAO(y)** | 54 (46-62) | 56 (50-62) | 0.102**^a^** |
| **Duration(y)** | 3 (2-6) | 3 (2-6) | 0.549**^a^** |
| **Smoking status** | 103 (30.0%) | 41 (26.1%) | 0.396**^b^** |
| **Taken anti-parkinsonsiam medicine or not** | 272 (79.3%) | 123 (78.3%) | 0.814**^b^** |
| **H-Y stage** | 2 (1.5-3) | 2 (1.5-3) | 0.769**^a^** |
| **UPDRS II, points** | 12 (9-17) | 12 (8-17) | 0.307**^a^** |
| **UPDRS III, points** | 27 (19-39) | 26 (18-34) | 0.466**^a^** |
| **Tremor, points** | 3 (1-5) | 3 (1-5) | 0.914**^a^** |
| **Rigidity, points** | 6 (3-9) | 5 (3-7) | **0.046^a^** |
| **Bradykinesia, points** | 9 (5-14) | 9 (6-14) | 0.930**^a^** |
| **Postural and gait abnormalities, points** | 4 (3-6) | 4 (2-6) | 0.322**^a^** |
| **PIGD /Intermediate /TD phenotype** | 201/33/109 | 88/16/53 | 0.866**^a^** |
| **Dyskinesia** | 43(12.5%) | 13(8.2%) | 0.173**^b^** |
| **MMSE** | 27 (25-29) | 28 (26-29) | **0.003^a^** |
| **TDI score** | 16.50 (11.50-19.50) | 24.75 (22.25-28.69) | **<0.001^a^** |
| **TD score** | 10.50 (6.50-13.50) | 16.25 (13.75-19.25) | **<0.001^a^** |
| **TI score** | 9.38 (6.25-12.75) | 16.25 (14.00-19.25) | **<0.001^a^** |
| **DI score** | 12 (9-15) | 18 (16-20) | **<0.001^a^** |
| **T score** | 3.50 (1.5-5.5) | 7.25 (5.5-9.5) | **<0.001^a^** |
| **D score** | 6.5 (4-8) | 9 (7-11) | **<0.001^a^** |
| **I score** | 6 (4-8) | 9 (8-10) | **<0.001^a^** |
