# Supplementary material for: The Discriminative Power of Different Olfactory Domains in Parkinson's Disease
Source: Front Neurol. 2020 Jun 2;11:420. doi: 10.3389/fneur.2020.00420 (PMC7280480; doi:10.3389/fneur.2020.00420)
Supplement: Supplementary file 2 [file Table_2.docx]

**Table S2. Multivariate linear regression analysis models for Olfactory Test Scores in Patients with PD**

| **Dependent variables** | **Significant variable*** | **B** | **Standardized β** | **t** | **P value** | **R^2^** | **R^2^ change** | **VIF** |
| --- | --- | --- | --- | --- | --- | --- | --- | --- |
| **a) TDI score** | Total |  |  |  | <0.001 | 0.160 |  |  |
|  | Constant | 18.635 |  | 4.955 | <0.001 |  |  |  |
|  | Age (years) | -0.213 | -0.262 | -6.003 | <0.001 |  | 0.111 | 1.106 |
|  | MMSE | 0.508 | 0.229 | 5.187 | <0.001 |  | 0.041 | 1.135 |
|  | Gender (male=1, female=0) | -1.479 | -0.092 | -2.186 | 0.029 |  | 0.008 | 1.032 |
| **b) Threshold score** | Total |  |  |  | <0.001 | 0.075 |  |  |
|  | Constant | 6.740 |  | 3.951 | <0.001 |  |  |  |
|  | Age (years) | -0.078 | -0.220 | -4.831 | <0.001 |  | 0.064 | 1.100 |
|  | MMSE | 0.105 | 0.109 | 2.389 | 0.017 |  | 0.011 | 1.100 |
| **c) Discrimination score** | Total |  |  |  | <0.001 | 0.152 |  |  |
|  | Constant | 8.095 |  | 5.199 | <0.001 |  |  |  |
|  | Age (years) | -0.093 | -0.278 | -6.336 | <0.001 |  | 0.115 | 1.106 |
|  | MMSE | 0.181 | 0.198 | 4.466 | <0.001 |  | 0.030 | 1.135 |
|  | Gender (male=1, female=0) | -0.573 | -0.087 | -2.046 | 0.041 |  | 0.007 | 1.032 |
| **d) Identification score** | Total |  |  |  | <0.001 | 0.111 |  |  |
|  | Constant | 4.880 |  | 3.099 | 0.002 |  |  |  |
|  | MMSE | 0.190 | 0.219 | 4.640 | <0.001 |  | 0.074 | 1.216 |
|  | Age (years) | -0.038 | -0.120 | -2.635 | 0.009 |  | 0.019 | 1.133 |
|  | UPDRS II | -0.048 | -0.105 | -2.301 | 0.022 |  | 0.010 | 1.136 |
|  | Gender (male=1, female=0) | -5.73 | -0.091 | -2.098 | 0.036 |  | 0.008 | 1.032 |

*Multiple linear regression analysis included those independent variables: age, sex, educational years, smoking status, disease duration, UPDRS-II points, UPDRS-III points, dyskinesia, MMSE, NMSS (cardiovascular dysfunction, sleep dysfunction, mood dysfunction, perceptual problems, gastrointestinal, urinary and sexual dysfunction). Variables with P value > 0.05 were excluded. The final model were reported here only with significant variates.

MMSE, Mini-Mental State Examination; UPDRS II, the Unified Parkinson’s Disease Rating Scale part II which evaluate the ability of daily living; B, unstandardized coefficients. R^2^ indicate that the model is able to explain how much variance in dependent variable. R^2^ change refer to the change of R^2^ value when the variable entered the equation of linear regression. VIF, variance inflation factor, they are no multicollinearity between the independent variables if VIF < 5.
